# Purinergic Vasotoxicity: Role of the Pore/Oxidant/K_ATP_ Channel/Ca^2+^ Pathway in P2X_7_-Induced Cell Death in Retinal Capillaries

**DOI:** 10.3390/vision2030025

**Published:** 2018-06-25

**Authors:** Maho Shibata, Eisuke Ishizaki, Ting Zhang, Masanori Fukumoto, Alma Barajas-Espinosa, Tong Li, Donald G. Puro

**Affiliations:** 1 Department of Ophthalmology & Visual Sciences, University of Michigan, Ann Arbor, MI 48105, USA; 2 Department of Molecular & Integrative Physiology, University of Michigan, Ann Arbor, MI 48105, USA

**Keywords:** K_ATP_ channels, oxidants, P2X_7_, pores, retina

## Abstract

P2X_7_ receptor/channels in the retinal microvasculature not only regulate vasomotor activity, but can also trigger cells in the capillaries to die. While it is known that this purinergic vasotoxicity is dependent on the transmembrane pores that form during P2X_7_ activation, events linking pore formation with cell death remain uncertain. To better understand this pathophysiological process, we used YO-PRO-1 uptake, dichlorofluorescein fluorescence, perforated-patch recordings, fura-2 imaging and trypan blue dye exclusion to assess the effects of the P2X_7_ agonist, benzoylbenzoyl-ATP (BzATP), on pore formation, oxidant production, ion channel activation, [Ca^2+^]_i_ and cell viability. Experiments demonstrated that exposure of retinal microvessels to BzATP increases capillary cell oxidants via a mechanism dependent on pore formation and the enzyme, NADPH oxidase. Indicative that oxidation plays a key role in purinergic vasotoxicity, an inhibitor of this enzyme completely prevented BzATP-induced death. We further discovered that vasotoxicity was boosted 4-fold by a pathway involving the oxidation-driven activation of hyperpolarizing K_ATP_ channels and the resulting increase in calcium influx. Our findings revealed that the previously unappreciated pore/oxidant/K_ATP_ channel/Ca^2+^ pathway accounts for 75% of the capillary cell death triggered by sustained activation of P2X_7_ receptor/channels. Elucidation of this pathway is of potential therapeutic importance since purinergic vasotoxicity may play a role in sight-threatening disorders such as diabetic retinopathy.

## 1. Introduction

Although functional P2X_7_ receptor/channels were discovered in retinal capillaries 15 years ago, much remains to be learned about their impact on retinovascular physiology and pathobiology. While it is clear that activation of P2X_7_ receptor/channels causes the abluminal pericytes of retinal capillaries to contract [1], the role of P2X_7_-induced vasoconstriction in regulating retinal blood flow is uncertain. One possibility is that these purinergic receptor/channels transduce the putative glial-to-vascular signal, ATP [2], into an alteration in local perfusion. They may also play a role in mediating vascular responses to nicotinamide adenosine dinucleotide (NAD^+^), which is candidate signaling molecule [3] whose ribosylation activates P2X_7_ receptor/channels in retinal capillaries [4].

Not only do P2X_7_ receptor/channels induce vasomotor activity, but we also discovered that their sustained activation can trigger the death of capillary cells [4,5,6]. Noteworthy is that this toxic effect of vasoactive purinergic input is conceptually similar to the extensively studied phenomenon of the neurotransmitter glutamate being neurotoxic. Previously, we reported that an essential mechanistic step in purinergic vasotoxicity is the formation of large transmembrane pores [4,5,6], whose opening during P2X_7_ activation has been intensively analyzed in many types of cells [7,8]. In non-vascular tissue, such as cells of the immune system, these pores appear to cause death by severely disrupting ionic gradients and/or by providing pathways for the egress of vital intracellular molecules [9,10,11,12]. However, since pore formation is relatively limited in retinal capillary cells [6], it remains uncertain how the opening of pores triggers death. Thus, the quest of this study was to identify events that occur downstream from pore formation and that play key roles in mediating purinergic vasotoxicity.

In this study using freshly isolated retinal microvascular complexes, we report that although the formation of P2X_7_ pores is required in order to trigger purinergic vasotoxicity in retinal capillaries, pore formation alone is not sufficient. Rather, a pore-dependent rise in intracellular oxidants is required. This study further revealed that the previously unappreciated pore/oxidant/K_ATP_ channel/Ca^2+^ pathway is the predominant mechanism mediating purinergic vasotoxicity in retinal capillaries.

## 2. Materials and Methods

Experimental protocols for animal use were approved by the Institutional Animal Care and Use Committee of the University of Michigan and were consistent with the guidelines of the Association for Research in Vision and Ophthalmology for use of animals in ophthalmic and vision research. Male Long-Evans rats were obtained from Charles River (Cambridge, MA, USA). At all times, animals were kept on a 12-h alternating light/dark cycle and received food and water *ad libitum*.

### 2.1. Microvessel Isolation

Using our previously described tissue print procedure [13], we isolated large complexes of microvessels from the retinas of adult rats. In brief, immediately after a rising concentration of carbon dioxide caused the death of a 6- to 14-week old rat, the retinas were removed and placed in solution A, which consisted of 140 mM NaCl, 3 mM KCl, 1.8 mM CaCl_2_, 0.8 mM MgCl_2_, 10 mM Na-Hepes, 15 mM mannitol, and 5 mM glucose at pH 7.4 with osmolarity adjusted to 310 mosmol L^−1^, as measured by a vapor pressure osmometer (Wescor, Inc., Logan, UT, USA). After adherent vitreous was removed with fine forceps, each retina was cut into quadrants and incubated for 22 to 26 min at 30 °C in 2.5 mL Earle’s balanced salt solution that was supplemented with 0.5 mM EDTA, 6 U papain (Worthington Biochemicals, Freehold, NJ, USA) and 2 mM cysteine; the pH was adjusted to approximately 7.4 by bubbling 5% carbon dioxide. After this incubation, the retinal pieces were transferred to a 60 mm Petri dish containing 5 mL of solution A, and one by one, each retinal quadrant was positioned with its vitreal surface up in a glass-bottomed chamber containing 1 mL of solution A. Subsequently, each retinal quadrant was gently sandwiched between the bottom of the chamber and a 15 mm diameter glass coverslip (Warner Instrument Corp., Hamden, CT, USA). After ~30 s, the coverslip was carefully removed; it contained adherent complexes of retinal microvessels. Microvascular complexes used in this study consisted of an arteriole encircled by “doughnut-shaped” myocytes, a tertiary vessel with ≥5 “dome-shaped” mural cell somas per 100 μm and a capillary network whose abluminal mural cells, the pericytes, appear as “bumps on a log” and have a density of ≤4 per 100 μm [13]. Photomicrographs of retinal microvessels isolated by this tissue print technique are available [13,14]. All measurements were obtained from the capillary portion. Experiments were completed within 7 h after microvessel isolation.

### 2.2. Imaging of Intracellular Oxidants

To detect intracellular oxidants, microvessels were exposed for 30 min to solution A supplemented with the oxidant-sensitive dye, 6-carboxy-2′,7′-dichlorodihydrofluorescein diacetate (carboxy-H_2_DCFDA; 5 µM; Invitrogen, Eugene, OR, USA). The microvessel-containing coverslip was then positioned in a 200 μL recording chamber, which was perfused at ~1.5 mL min^−1^ via a gravity-fed system. Subsequent exposure to dye-free solution A for 10 min allowed carboxy-H_2_DCFDA to be cleaved by intracellular esterases to carboxy-H_2_DCF, which upon oxidation becomes fluorescent dichlorofluorescein (DCF). Microvessels were observed using a Nikon Eclipse TE300 microscope at X400 with a 40X water-immersion objective. The light source was a high intensity mercury lamp coupled to an Optoscan Monochromator (Cairn Research Ltd., Faversham, UK). Fluorescence was detected with excitation and emission wavelengths of 490 and 520 nm, respectively. Digital imaging of DCF fluorescence was performed using an optical sensor (Sensicam, Cooke Corp., Auburn Hills, MI, USA). Axon Imaging Workbench software (MDS Analytical Technologies) facilitated control of the imaging equipment and the collection of data. To minimize photo-oxidation, illumination was limited to 400-ms exposures at 30 s to 90 s intervals. Autofluroescence was not detected in microvessels not been exposed to carboxy-H_2_DCFDA. In microvascular complexes exposed to carboxy-H_2_DCFDA, both mural cells and endothelial cells became loaded with this dye. No attempt was made to selectively detect fluorescence in mural or endothelial cells; fluorescence from both cell types was detected. Regions of interest (ROIs) were selected on microvessels and in order to measure background fluorescence, on cell-free areas of the coverslip. Subtraction of background fluorescence from the intensity of fluorescence measured in microvascular ROIs yielded the net fluorescence. If F_DCF_ varied by >5% during a 200-s control period in solution A, the microvascular was not further studied. For each group of ROIs, the control value was the average net fluorescence during the 200-s period prior to the onset of exposure to the P2X7 agonist, benzoylbenzoyl-ATP (BzATP, 100 µM). Net fluorescence measurements were plotted in Figure 2A as the percent of the control value. In Figures 2B and 4, net fluorescence increases were measured 600 s after the onset of BzATP exposure. In experiments using A740003 (200 nM), UTP (30 µM), apocynin (300 µM), n-acetyl-cysteine (NAC, 100 µM), glibenclamide (0.5 µM) or low calcium solution A (solution A without added CaCl_2_), pre-incubations prior to exposure to BzATP were at room temperature for 15 min, 30 min, 30 min, 60 min, 10 min and 30 min, respectively.

### 2.3. YO-PRO-1 Uptake

To detect capillary cells containing transmembrane pores, microvascular complexes were exposed to propidium di-iodide dye, YO-PRO-1 (Molecular Probes, Eugene, OR, USA), which is a 629 Da molecule that becomes fluorescent after entering a cell and binding to nucleic acids. Initially, microvessels were exposed for 1 h at room temperature to solution A in the absence or in the presence of 30 µM UTP, 300 µM apocynin or 0.5 µM glibenclamide. Subsequently after addition of 100 µM BzATP to some experimental groups, microvessels were incubated for 4 h at 37 °C and 100% humidity. Immediately after this incubation, bathing solutions were supplemented with 5 µM YO-PRO-1, and incubation was continued at 37 °C for 30 min. After washout of the YO-PRO-containing solutions, microvessel-containing coverslips were positioned in a chamber located on the stage of a Nikon Eclipse TE300 or E800 microscope (Nikon, Tokyo, Japan) equipped for fluorescence and using X20 objectives. Differential interference contrast optics facilitated detection of cells lacking fluorescence. For each coverslip, more than 70 microvascular cells were examined, and the percentage of YO-PRO-positive cells was determined. It did not prove feasible to subclassify cells as endothelial or abluminal. The BzATP-induced increase in YO-PRO positive capillary cells was calculated by subtracting the percentage of positive cells observed in the appropriate control group from the BzATP-containing group. Notable is that in the absence of BzATP, neither 200 nM A740003, 30 µM UTP, 100 µM NAC, 0.5 µM glibenclamide nor the low calcium solution A significantly affected the percentage of YO-PRO positive cells, which in solution A alone was 9.9 ± 0.9% (*n* = 21) after 4 h at 37 °C.

### 2.4. Cell Viability Assay

Microvascular cells that failed to exclude trypan blue were classified as dead. Microvessel-containing coverslips were exposed to 0.04% trypan blue assay in solution A for 15 min, and the percentage of trypan blue positive cells was determined by examining microvessel-containing coverslips at X100 magnification with an inverted microscope equipped with bright-field optics. Of note, because trypan blue-containing cells typically were swollen, identification of these cells as being endothelial or abluminal was uncertain, and thus, sub-classification of microvascular cells into these two types was not done in the cell death assays. As reported previously [6], the validity of this 961-Da vital stain was not compromised by the formation of P2X_7_ pores, which are permeable to molecules of ≤900 Da [15]. Prior to the onset of experimental conditions, cell viability was determined in microvascular complexes whose locations on each coverslip were carefully documented so that cell death could be re-assessed in the identical microvascular region. At least 150 microvascular cells per coverslip were counted. In experiments using A740003, UTP, apocynin, n-acetyl-cysteine or glibenclamide, microvessels were exposed to the additive in solution A for 1 h at room temperature. Subsequently, after the addition of BzATP, microvascular complexes were maintained for 6 h at 37 °C and 100% humidity. Immediately after this incubation, the percentage of trypan blue capillary cells was again assayed for each of the microvascular regions assessed at time zero. The increase in cell death between time 0 and at 6 h was then calculated. Because the percentage of trypan blue capillary cells maintained for 6 h at 37 °C increased from 4.4 ± 0.3% to 5.5 ± 0.2% (*n* = 23), the amount of cell death induced by BzATP was calculated by subtracting this 1.1% increase from the increase in trypan blue positive cells measured during BzATP exposure.

### 2.5. Electrophysiology

The perforated-patch configuration of the patch clamp technique was used to detect ionic currents in microvascular complexes located on coverslips positioned in a recording chamber (*V* = 0.5 mL), which was perfused (~1.5 mL min^−1^) with solution A, which could be supplemented with 100 µM BzATP without or with 0.5 µM glibenclamide. Experiments were at 22 °C to 23 °C. Recording pipettes that were filled with a solution consisting of 50 mM KCl, 65 mM K_2_SO_4_, 6 mM MgCl_2_, 10 mM K-Hepes, 60 μg mL^−1^ amphotericin B and 60 μg mL^−1^ nystatin at pH 7.4 with the osmolarity adjusted to 280 mosmol L^−1^ and that had resistances of 5 to 10 MΩ were mounted in the holder of a patch-clamp amplifier (Axopatch 200B, MDS Analytical Technologies, Union City, CA, USA) and were positioned onto the surface of capillary pericytes, which are easily identified by their “bump on the log” location [13,16]. Recordings with a seal resistance of ≥10 GΩ seal and an access resistance of <25 MΩ were used. Currents were filtered with a four-pole Bessel filter, digitally sampled and stored on a computer equipped with pClamp 10 (MDS Analytical Technologies) and Origin graphics software (OriginLab, Northampton, MA, USA). Notable is that ~95% of the current monitored via a perforated-patch pipette sealed onto a retinal capillary pericyte is generated by cellular neighbors that are in electrotonic communication with the sampled cell [17,18,19]. Indicative that the space-clamp of the perforated-patch recordings was adequate, the observed reversal potential for the glibenclamide-sensitive current was close to the calculated equilibrium potential of −103 mV for K+ (Figure 3). Current-voltage relations were established by pCLAMP-controlled voltage protocols and consisted of either steps of voltage or a negative-to-positive ramping (66 mV/s) of voltage from a holding potential of −58 mV. Using the I-V plot that is the difference between the I-V relations in the absence and presence of glibenclamide, the K_ATP_ conductance was determined by calculating the average of the conductances measured at 10-mV intervals, from −118 mV to +22 mV. The zero-current potential was defined as the membrane potential.

### 2.6. Calcium Imaging

Freshly isolated retinal microvascular complexes were incubated in solution A supplemented with 5 μM fura-2AM (Molecular Probes) at 37 °C for 90 min. After allowing the AM ester to be cleaved, a coverslip containing fura-loaded microvessels was positioned in a 200 μL recording chamber perfused at ~1.5 mL min^−1^ with solution A, which as described in the results section was supplemented with 100 µM BzATP and subsequently also with 0.5 µM glibenclamide. Digital imaging was performed using a Nikon Eclipse E600 FN microscope, an optical sensor (Sensicam, Cooke Corp.) and a high intensity mercury lamp coupled to an Optoscan Monochromator (Cairn Research Ltd., Faversham, UK); imaging equipment and data collection were controlled using MetaFluor software (Molecular Devices, Sunnyvale, CA, USA). As noted previously, the pericytes on retinal capillaries load well with fura-2 while little of this fluorescent dye enters the endothelial cells [20]. Regions of interest (ROIs) encircled pericyte somas. Visualization of microvessels with the aid of differential interference contrast microscopy was used to help confirm that ROIs were of abluminal cell somas. Isolated microvascular complexes lacked detectable autofluorescence. Fluorescent intensities within ROIs were measured at 340 nm and 380 nm at a sampling rate of 0.1 Hz. As detailed earlier, Mn^2+^ quenching not routinely performed because only a minimal amount of fluorescence in isolated retinal microvascular is derived from fura-2 that is not sensitive to free Ca^2+^ [14]. Background fluorescence of the optical system was measured in each experiment by placing some ROIs in cell-free areas of the coverslip. After subtracting this background, the F_340_/F_380_ ratio was calculated and converted to the intracellular calcium concentration by use of a standard equation [21] in which R_min_ and R_max_ were determined as detailed previously [14]. For each microvessel monitored, the pericyte calcium concentration for each experimental condition was the mean measured during a 1- to 3-min period (10 to 30 sample points). The data summarized in Figure 4B were obtained prior to exposure to BzATP and 5 min after the onset of exposure to BzATP without or with 0.5 µM glibenclamide.

### 2.7. Chemicals

Unless otherwise noted, chemicals were obtained from Sigma (St. Louis, MO, USA).

### 2.8. Statistics

Data are given as means ± SE with *n* being the number of cells sampled. Unless noted otherwise, probability was evaluated by Student’s two-tailed *t*-test, unpaired or paired as appropriate. *p* > 0.05 indicated failure to detect a significant difference. For greater than two groups, an analysis of variance was performed using commercially available software (Origin 2017) with the subsequent application of the Bonferroni correction.

## 3. Results

**Figure 1:**
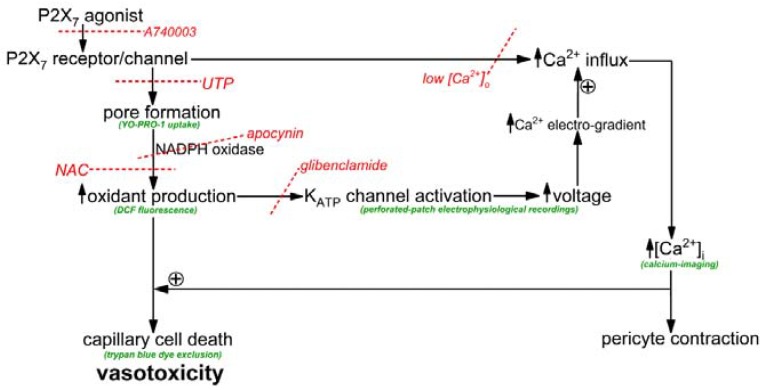
Overview of the conceptual framework of this experimental study. Assays are shown in green and inhibitors are in red with the dotted lines indicating sites of inhibition. UTP inhibits pore formation by a mechanism involving P2Y_4_ receptors and phospholipase [5]. Low [Ca^2+^]_o_ was solution A without added calcium. NAC is the antioxidant, n-acetyl-cysteine. Although not the focus of this study, capillary vasoconstriction occurs with P2X_7_ activation [1,26].

The objective of this study was to elucidate how the activation of P2X_7_ receptor/channels can cause cells in retinal capillaries to die. To guide experimentation, we built upon previous studies to formulate a working model. As shown in Figure 1, the key instigating event in purinergic vasotoxicity is the formation of large transmembrane pores during sustained P2X_7_ activation [5]. Further, studies of non-retinal cells have demonstrated that the opening of P2X_7_ pores increases intracellular oxidants by a mechanism involving NADPH oxidase [22,23]. In addition, based on our previous analysis of the electrophysiological effects of the oxidant H_2_O_2_ [24], we postulated that the P2X_7_-induced increase in oxidants may activate redox-sensitive K_ATP_ channels whose voltage-increasing effect would enhance the electro-gradient for the influx of calcium whose elevated intracellular concentration is known to increase the lethality of oxidative stress [24,25]. Taken together, these considerations led us to posit that a pore/oxidant/K_ATP_ channel/Ca^2+^ pathway boosts the vulnerability of retinal capillaries to P2X_7_ vasotoxicity (Figure 1).

**Figure 2:**
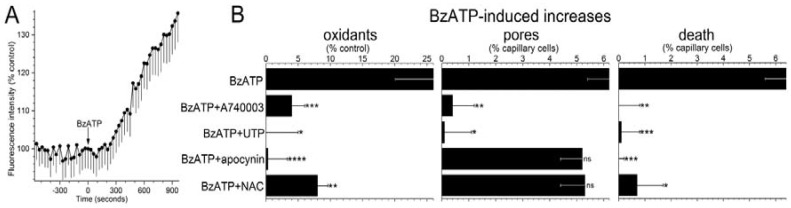
Effect of the P2X_7_ agonist, BzATP, on intracellular oxidants, transmembrane pores and cell viability in capillaries located within microvascular complexes freshly isolated from the rat retina. (**A**) Fluorescence intensity of the oxidation-sensitive dye, dichlorofluorescein (DCF), before and during exposure to 100 µM BzATP. Each data point is the mean of 9 regions of interest in retinal capillaries. Fluorescence intensities (F_DCF_) are plotted as the percentage of the control value. (**B**) Effect of various additives on the increase in F_DCF_ induced by a 600-s exposure to 100 µM BzATP. Additives: A740003 (200 nM), an inhibitor of P2X_7_ receptors; UTP (30 µM), a nucleotide whose effect on retinal microvessels includes inhibition of P2X_7_ pore formation; apocynin (300 µM), an inhibitor of NADPH oxidase, and NAC (n-acetyl cysteine, 100 µM), an antioxidant. Sample size: 47 ± 8. * *p* = 0.0081; ** *p* = 0.0052; *** *p* = 0.0009; **** *p* = 0.0003 (**Left panel***).* Effect of the additives on the BzATP-induced increase in the percentage of capillary cells containing the 629 Da dye, YO-PRO-1, whose entry is indicative of the presence of transmembrane pores. Sample size: 14 ± 6. * *p* = 0.0014; ** *p* < 0.0001; ns, not significantly different (**Middle panel**). Effect of the additives on BzATP-induced capillary cell death. Sample size: 12 ± 5. * *p* = 0.0005; ** *p* = 0.0001; *** *p* < 0.0001 (**Right panel**).

To begin to assess the role of oxidants in purinergic vasotoxicity, the fluorescence of dichlorofluorescein (DCF) was used to detect a change in capillary cell oxidants during exposure to the P2X_7_ agonist, BzATP (100 µM). As illustrated in Figure 2A and summarized in Figure 2B, BzATP induced a significant increase in F_DCF_. Additional DCF experiments were performed in the presence of 200 nM A740003, which is a P2X_7_ receptor antagonist [27] and 30 µM UTP, whose activation of P2Y_4_ inhibits P2X_7_ pore formation in retinal microvessels [5]. We found that both A74003 and UTP markedly attenuated BzATP-induced formation of pores and rise in capillary cell F_DCF_ (Figure 2B). Indicative the BzATP-induced oxidant production is not a rapidly reversible process, we observed that F_DCF_ increased by 9 ± 2% (*n* = 9; *p* = 0.0158) during the initial 5 min after the washout of BzATP. Taken together, these results led us to conclude P2X_7_ activation triggers a sustained boost in the level of oxidants in retinal capillary cells.

Supporting a role for NADPH oxidase in mediating the increase in oxidants during P2X_7_ activation, the BzATP-induced increase in capillary DCF fluorescence was effectively prevented (*p* = 0.0009) by the inhibitor of this enzyme, apocynin (300 µM; Figure 2B, left panel). Furthermore, consistent with NADPH oxidase and oxidants acting downstream from pore formation, the BzATP-induced increase in YO-PRO-positive cells (Figure 2B, middle panel) was not significantly affected by either apocynin or the antioxidant, n-acetyl cysteine (NAC, 100 µM), which markedly limited the P2X_7_-induced rise in capillary cell oxidants (Figure 2B, left panel).

Indicative that purinergic vasotoxicity is mediated via a pathway involving P2X_7_ receptors, pores, NADPH oxidase and oxidants, capillary cell death induced by BzATP was markedly attenuated by A740003, UTP, apocynin and NAC (Figure 2B, right panel). Taken together, these results support a model in which pore-dependent oxidative stress plays a key role in triggering the death of retinal capillary cells during the sustained activation of their P2X_7_ receptors.

**Figure 3:**
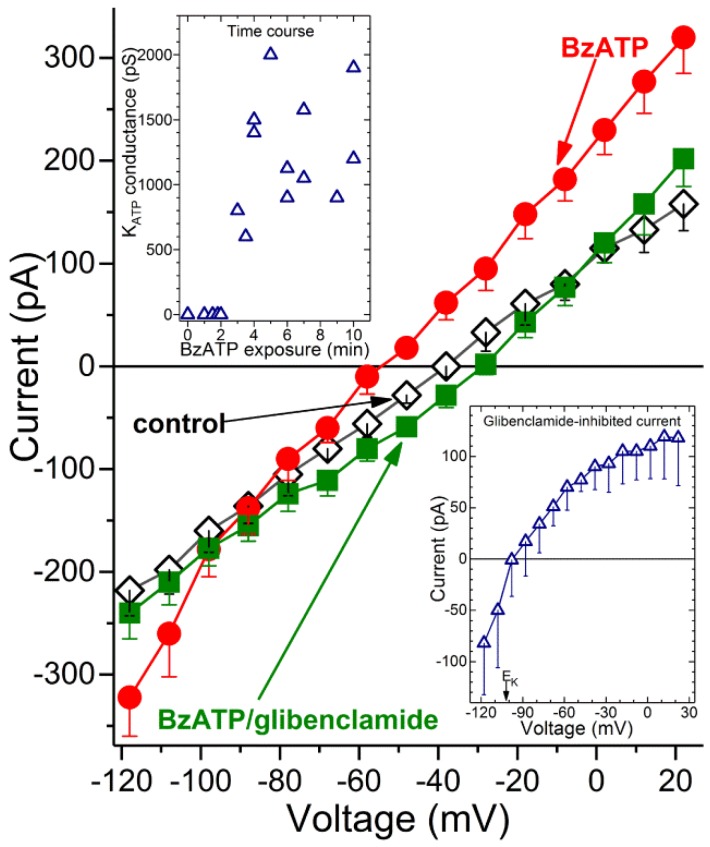
Detection of a K_ATP_ conductance in capillaries during sustained P2X_7_ activation. Averaged I-V plots (*n* = 4) obtained in solution A without additives (control), in the presence of 100 µM BzATP and after addition of 0.5 µM glibenclamide. (**Lower inset**), The I-V relations of the glibenclamide-sensitive current. E_K_, K^+^ equilibrium potential. (**Upper inset**), Time course for the BzATP-induced activation of the glibenclamide-sensitive conductance. The zero-time point represents 12 recordings, while the other data points are from single recordings, which are plotted at their mean sampling times.

With earlier studies demonstrating that retinal capillaries express redox-sensitive K_ATP_ channels [24,28,29,30], we posited that the increase in oxidants during P2X_7_ activation may in turn, result in the activation of K_ATP_ channels. To begin to assess this possibility, ionic currents were monitored via perforated-patch pipettes sealed onto pericytes located on the abluminal surface of capillaries located within freshly isolated retinal microvascular complexes. Figure 3 shows the averaged I-V plots obtained from recordings in which microvessels were initially bathed in solution A (◊), then exposed for 3 to 10 min to BzATP-containing solution A and finally ~2 min after the addition of the K_ATP_ channel blocker, glibenclamide (0.5 µM). BzATP exposure was associated with the development of a hyperpolarizing current (Figure 3, ●). Indicative that the BzATP-induced conductance was due to the opening of K_ATP_ channels, this hyperpolarizing current was inhibited by glibenclamide (Figure 3, ■). As expected for a K_ATP_ conductance, the I-V relations of the glibenclamide-sensitive current exhibited inward rectification and had a reversal potential close to the equilibrium potential for K^+^, i.e., −103 mV (Figure 3, lower inset). Indicative that BzATP results in K_ATP_ channel activation by a mechanism involving P2X_7_ receptors, we did not detect (*n* = 3) a hyperpolarizing current during 3 to 10 min of BzATP exposure in the presence of the specific P2X_7_ blocker, A740003 (200 nM). In a series of 12 recordings obtained from retinal capillaries during 3 to 10 min of exposure to BzATP, the mean K_ATP_ conductance was 1250 ± 130 pF, and the resting membrane potential voltage was −65 ± 3 mV. In contrast, retinal capillaries bathed in solution A without BzATP did not exhibit a detected glibenclamide-sensitive current (*n* = 12; Figure 3, upper inset), and the resting voltage was potential of −41 ± 2 mV. These findings demonstrate that a hyperpolarizing K_ATP_ current is activated during the sustained activation of P2X_7_ receptor/channels.

What is the time course for the activation of K_ATP_ channels during exposure to the P2X_7_ agonist? As shown in the upper inset of Figure 3, a glibenclamide-inhibited current was not detected during the initial ~2 min of BzATP exposure. Of note, we have detailed elsewhere [1,5] that during the initial 2 min of exposure to this agonist, the opening of P2X_7_ channel/pores generates a depolarizing current. Subsequently, at ~3 min of BzATP exposure, a K_ATP_ conductance is activated (Figure 3, upper inset). This hyperpolarizing current completely counterbalances the depolarizing current generated by the flux of cations through the P2X_7_ channel/pores. We also found that a K_ATP_ conductance persists during sustained P2X_7_ activation. Namely, at 30 to 60 min of BzATP exposure, the glibenclamide-sensitive conductance was 1385 ± 135 pS (*n* = 4), which was not significantly different than the K_ATP_ conductance observed at 3 to 10 min. Taken together, our electrophysiological recordings establish that after a ~2-min lag, the sustained activation of P2X_7_ receptor/channels results in the opening of K_ATP_ channels whose hyperpolarizing action drives the membrane potential of capillaries markedly more negative.

**Figure 4:**
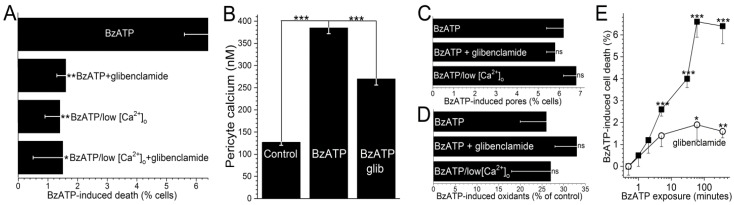
The protective effect of glibenclamide on P2X_7_-induced capillary cell death. (**A**) Capillary cell death induced during a 6-h exposure to 100 µM BzATP in the absence or presence of 0.5 µM glibenclamide; these chemicals were in either solution A or a modification of solution A lacking added calcium. Glibenclamide significantly diminished BzATP-induced cell death in solution A, but not in low calcium. Sample sizes: 15 ± 6. * *p* = 0.0052; ** *p* < 0.0001. (**B**) Pericyte calcium in capillaries exposed for 5 min to 100 µM BzATP without or with 0.5 µM glibenclamide. Sample sizes: 194 ± 46. *** *p* < 0.0001. (**C**) Lack of a significant effect of 0.5 µM glibenclamide or low extracellular calcium on YO-PRO-1 uptake during a 4-h exposure to 100 µM BzATP. Sample sizes: 15 ± 7. (**D**) Lack of a significant effect of glibenclamide (0.5 µM) or low calcium on the increase in F_DCF_ observed during a 600-s exposure to 100 µM BzATP. Sample sizes: 36 ± 10. (**E**) Effect of the duration of 100 µM BzATP exposure in the absence or presence of 0.5 µM glibenclamide on capillary cell viability assayed 6 h after the onset of BzATP exposure. Sample sizes for the glibenclamide-free and glibenclamide-containing protocols were 11 ± 4 and 16 ± 7, respectively. * *p* = 0.0494; ** *p* = 0.0143, *** *p* < 0.0001.

Consistent with the oxidants playing a key role in activating K_ATP_ channels during P2X_7_ activation, we found that pre-treatment of retinal microvessels with the antioxidant, NAC (100 µM), prevented the development of the BzATP-induced hyperpolarizing current. Namely, with a >5-min exposure to BzATP in NAC-containing solution A, no glibenclamide-sensitive current was detected (*n* = 4). Thus, our electrophysiological experiments support a scenario in which the pore-dependent increase in oxidants results in the activation of K_ATP_ channels. Indicative that the pathophysiological importance of the K_ATP_ channel activation, glibenclamide decreased BzATP-induced capillary cell death by 75 ± 8% (*p* < 0.0001; Figure 4A). It is notable that while cell death is enhanced by K_ATP_ channel activation during BzATP exposure, we have previously demonstrated that the activation of these channels under control conditions does not affect capillary cell viability [31]. Taken together, our observations show that the opening of K_ATP_ channels during sustained P2X_7_ receptor activation boosts purinergic vasotoxicity.

As illustrated in Figure 1, our working hypothesis is that by increasing the electro-gradient for calcium influx, the oxidant-induced opening of hyperpolarizing K_ATP_ channels enhances calcium influx via P2X_7_ channels/pores and thereby, increases capillary cell calcium. In agreement with this scenario, calcium-imaging experiments demonstrated that glibenclamide significantly (*p* < 0.0001) attenuated the BzATP-induced increase in pericyte calcium (Figure 4B).

Furthermore, indicative of the importance of calcium influx in mediating purinergic vasotoxicity, P2X_7_-induced capillary cell death was markedly diminished when microvessels were exposed to an extracellular solution lacking added calcium (Figure 4A). This dependence on extracellular calcium is consistent with our proposal (Figure 1) that the oxidant-induced activation of hyperpolarizing K_ATP_ channels boosts P2X_7_-induced cell death by increasing calcium influx and thereby, increasing the intracellular rise in calcium, which is known to enhance to lethality of oxidative stress in capillary cells [24]. Furthermore, consistent with K_ATP_ channels boosting BzATP-induced death by a mechanism dependent on calcium influx, the protective effects of glibenclamide and low [Ca^2+^]_o_ were not additive (Figure 4A). Also in agreement with our working model (Figure 1) that K_ATP_ channel activation and increased calcium influx are events occurring downstream from the formation of pores and the production of oxidants, neither glibenclamide nor low extracellular calcium significantly affected the increases in YO-PRO-1 uptake (Figure 4C) and DCF fluorescence (Figure 4D) induced by BzATP exposure.

To further characterize purinergic vasotoxicity in retinal capillaries, we sought to establish the relationship between the duration of P2X_7_ activation and the amount of induced cell death. As shown in Figure 4E, a ≤2-min exposure to BzATP failed to induce significant cell death when assayed 6 h later. On the other hand, a 5-min exposure did result in significant (*p* < 0.0001) subsequent capillary cell death (Figure 4E). As shown in Figure 4E, ~75% of the BzATP-induced cell death triggered by a ≥30-min exposure to the P2X_7_ agonist was mediated by a glibenclamide-sensitive, i.e., K_ATP_-dependent, mechanism.

Taken together, the results of this investigation provide strong experimental support for our working hypothesis that sustained P2X_7_ activation results in a pore-dependent and NADPH oxidase-driven increase in oxidants. In turn, oxidants drive the activation of K_ATP_ channels whose hyperpolarizing effect enhances the influx of calcium whose elevated intracellular concentration boosts the lethality of oxidative stress.

## 4. Discussion and Conclusions

This study provides new insights into the mechanism by which the activation of P2X_7_ purinergic receptors triggers vasotoxicity in retinal capillaries. The experimental results support our working model (Figure 1) in which the formation of transmembrane pores during P2X_7_ receptor/channel activation is necessary, but not sufficient to cause purinergic vasotoxicity. Rather, an essential pathophysiological step is the pore-driven, NADPH oxidase-dependent generation of capillary cell oxidants. A key consequence of this rise in oxidants is the activation of redox-sensitive K_ATP_ channels, which by driving the membrane potential more negative, increase the electro-gradient for influxing calcium whose elevated intracellular concentration markedly enhances the lethality of oxidative stress. Our analysis reveals that the previously unappreciated pore/oxidant/K_ATP_ channel/Ca^2+^ pathway accounts for ~75% of cell death induced in retinal capillaries by the sustained activation of P2X_7_ receptor/channels.

The question arises as to when purinergic vasotoxicity may occur in the retina. We posit that because capillary cell death is virtually nonexistent in the normal retina [32], the pore/oxidant/K_ATP_ channel/Ca^2+^ pathway must be tightly regulated. Consistent with this, the vasoactive molecule, ATP, activates not only P2X_7_ receptor/channels, but also P2Y_4_ receptors, which via a phospholipase-dependent mechanism potently inhibit the formation of P2X_7_ pores [5]. In this way, extracellular ATP may not only serve as a vasoactive signal, but may also play a vasoprotective role. In contrast, ribosylation reactions involving the putative extracellular signal, NAD^+^ [3] result in the selective activation of P2X_7_ receptor/channels and the formation of pores that can trigger capillary cell death [4]. However, it is likely that NAD^+^-induced vasotoxicity is normally prevented by ecto-NAD^+^-glycohydrolase C38, which an NAD^+^-catabolizing enzyme located on the surface of the glia that fully ensheath the retinovasculare [3]. On the other hand, because the capillaries in the diabetic retina are ~100-fold more vulnerable to P2X_7_-induced vasotoxicity [4,6], an important topic for future investigation is the impact of diabetes on the pore/oxidant/K_ATP_ channel/Ca^2+^ pathway.

The capillaries of the retina appear to be particularly vulnerable to purinergic vasotoxicity due to their abundance of K_ATP_ channels and, unlike nearly all other sites within the circulatory system, dearth of functional voltage-dependent calcium channels (VDCCs) [14]. While this ion channel profile is likely to be a specialized physiological adaptation to help meet the unique challenges faced by the retinal vasculature [13], the impact of K_ATP_ channel activation on intracellular calcium is fundamentally altered. Namely, in vessels possessing substantial VDCCs, a K_ATP_-induced hyperpolarization deactivates these calcium channels and thereby, causes cell calcium to decrease. This decrease in calcium plays a key role in the cell protective effect of K_ATP_ channel activation observed many vascular and non-vascular tissues [33]. Conversely, in the VDCC-sparse retinal capillaries, the activation of K_ATP_ channels results in a rise in calcium as hyperpolarization enhances the electro-gradient for Ca^2+^ influx via the calcium-permeable nonspecific cation channels [24], which are expressed in these microvessels and whose activity is not regulated by voltage [34]. Taken together, our studies demonstrate the vulnerability of retinal capillaries purinergic vasotoxicity, as well as to hypoxia [31] and oxidative stress [24], is markedly boosted by the K_ATP_-driven increase cell calcium.

Our conclusions concerning the role of pore/oxidant/K_ATP_ channel/Ca^2+^ pathway in P2X_7_-induced vasotoxicity are based on experiments using microvascular complexes freshly isolated from the rat retina. One benefit of studying microvessels in isolation is that potentially confounding effects mediated by nonvascular retinal cells are eliminated. In addition, it is possible to precisely control the concentrations of agonists and inhibitors, as well as the duration of exposure to these chemicals. However, our characterization of the P2X_7_ pore/oxidant/K_ATP_ channel/Ca^2+^ pathway in isolated microvessels will require in vivo validation, although the types of fluorescence-imaging and patch-clamping assays used in this study have yet to be performed in capillaries in oculo. Additional study is also needed to characterize the specific type(s) of K_ATP_ channels expressed in retinal capillaries and involved in boosting purinergic vasotoxicity. An additional caveat includes the need for caution when extrapolating findings obtained with rodent retinal microvessels to the microvasculature of the human retina. Yet, despite caveats, the experimental approach used in this study has revealed a previously unappreciated pathway that potently boosts the vulnerability of retinal capillaries to purinergic vasotoxicity.

In summary, the pore/oxidant/K_ATP_ channel/Ca^2+^ pathway accounts for ~75% of the vasotoxicity triggered by the sustained activation of P2X_7_ receptor/channels in retinal capillaries. A hope for the future is that elucidation of the pathophysiological role of this pathway will provide targets for novel therapeutic strategies to prevent cell death in retinal capillaries.
